# Supplementary material for: Fasting and Postprandial DNA Methylation Signatures in Adipose Tissue from Asymptomatic Individuals with Metabolic Alterations
Source: Int J Mol Sci. 2025 Nov 22;26(23):11306. doi: 10.3390/ijms262311306 (PMC12692098; doi:10.3390/ijms262311306)
Supplement: Supplementary file 1 [file ijms-26-11306-s001.zip › Supplementary Figures.pdf]

Supplementary Figures

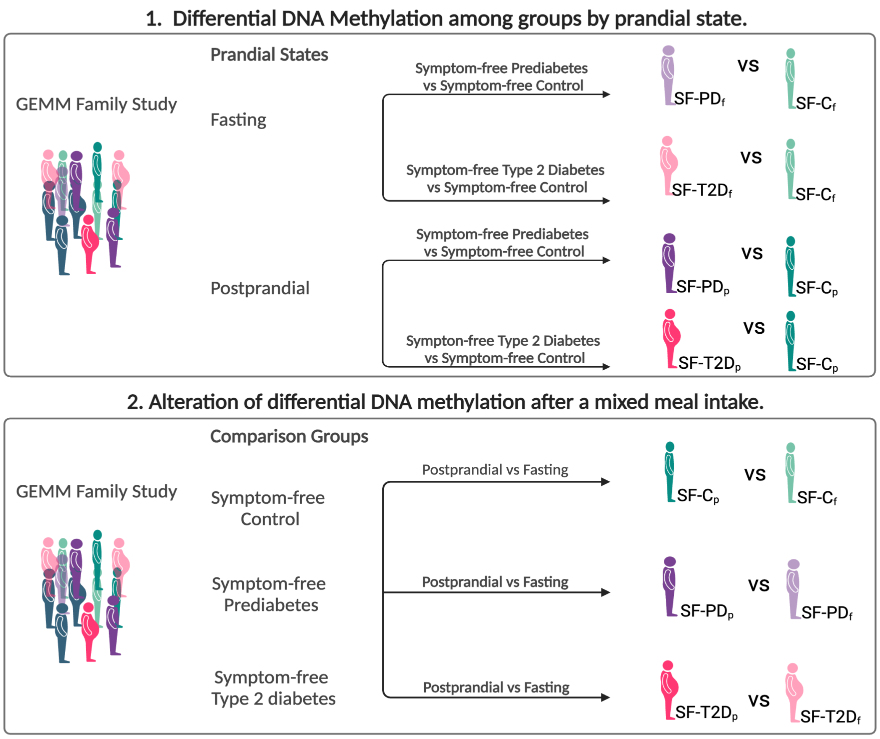

Supplementary Fig. 1. Overview of the DNA methylation analysis by group and prandial state. SF, Symptom-free; Cf, control group-fasting; PDf, prediabetes group-fasting; T2Df, type 2 diabetes group-fasting; Cp, control group-postprandial; PDp, prediabetes group-postprandial; T2Dp, type 2 diabetes group-postprandial.

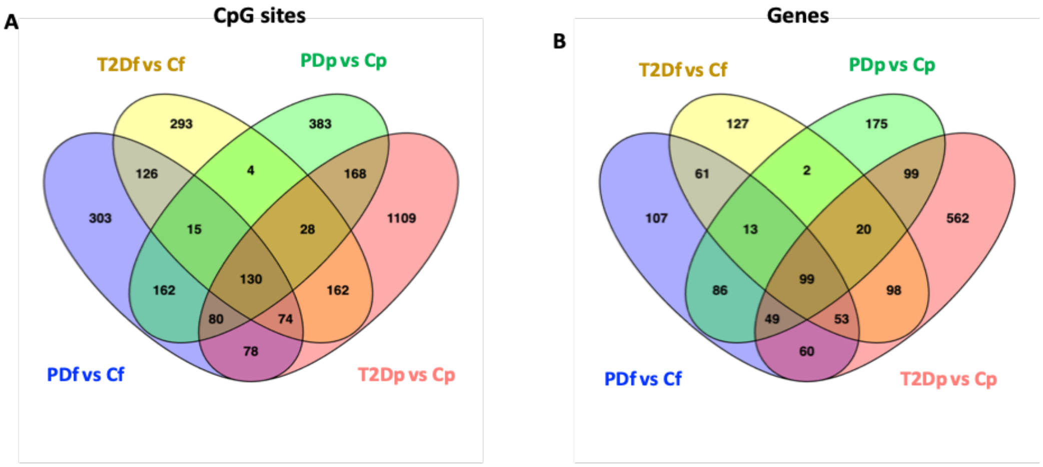

Supplementary Fig. 2. Venn diagrams of methylation differences between contrasts. The 4-way Venn diagrams show the overlap of differentially methylated CpGs (A) and differentially methylated genes (B) between comparisons by prandial state. All groups are compared to the control group. Cf, control group-

fasting; PDf, prediabetes group-fasting; T2Df, type 2 diabetes group-fasting; Cp, control group-postprandial; PDp, prediabetes group-postprandial; T2Dp, type 2 diabetes group-postprandial.

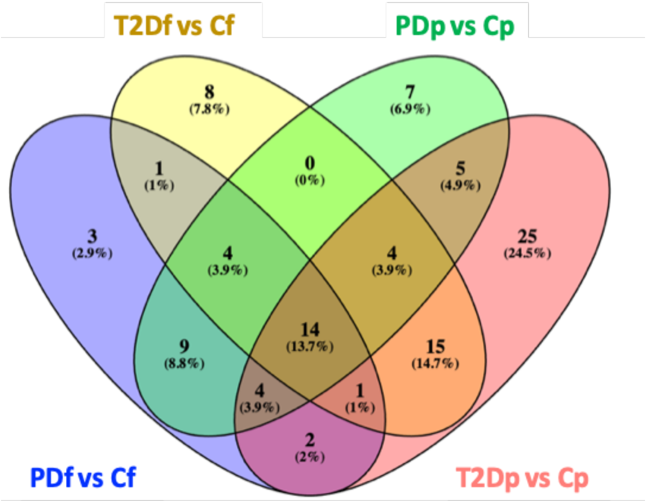

Supplementary Fig. 3. Venn diagram showing KEGG pathways shared between groups and prandial states. Cf, control group-fasting; PDf, prediabetes group-fasting; T2Df, type 2 diabetes group-fasting; Cp, control group-postprandial; PDp, prediabetes group-postprandial; T2Dp, type 2 diabetes group-postprandial.
